# Silver and Gold Nanoparticles Alter Cathepsin Activity In vitro

**DOI:** 10.1007/s11671-010-9746-3

**Published:** 2010-08-29

**Authors:** Janice L Speshock, Laura K Braydich-Stolle, Eric R Szymanski, Saber M Hussain

**Affiliations:** 1 Applied Biotechnology Branch, Human Effectiveness Directorate, 711th Human Performance Wing, Air Force Research Laboratory (711 HPW/RHPB), Wright-Patterson Air Force Base (AFB), Area B, R ST, Bldg 837, Dayton, OH 45433-5707, USA; 2 Electrical and Computer Engineering Department, College of Engineering, The Ohio State University, Columbus, OH USA

**Keywords:** Cathepsin, Protease, Nanoparticle, Gold, Silver

## Abstract

Nanomaterials are being incorporated into many biological applications for use as therapeutics, sensors, or labels. Silver nanomaterials are being utilized for biological implants and wound dressings as an antiviral material, whereas gold nanomaterials are being used as biological labels or sensors due to their surface properties and biocompatibility. Cytotoxicity data of these materials are becoming more prevalent; however, little research has been performed to understand how the introduction of these materials into cells affects cellular processes. Here, we demonstrate the impact that silver and gold nanoparticles have on cathepsin activity in vitro. Cathepsins are important cellular proteases that are imperative for proper immune system function. We have selected to examine gold and silver nanoparticles due to the increased use of these materials in biological applications. This manuscript depicts how both of these types of nanomaterials affect cathepsin activity, which could impact the host's immune system and its ability to respond to pathogens. Cathepsin B activity decreases in a dose-dependent manner with all nanoparticles tested. Alternatively, the impact of nanoparticles on cathepsin L activity depends greatly on the type and size of the material.

## Introduction

Cathepsins are lysosomal proteases that are present in many different types of mammalian cells. Cathepsins B and L are cysteine proteases localized in the lysosomes and endosomes and are mainly involved in protein degradation and antigen presentation [1]. Cathepsin B is mainly expressed in antigen-presenting cells (APCs) and is involved in protein processing for presentation and is responsible for the degradation of the invariant chain (Ii), a chaperone molecule essential for major histocompatibility complex (MHC) class II molecules assembly and transport [2,3]. Cathepsin B also functions to process MHC-II binding peptides in the endosome [3]. In addition to antigen presentation, cathepsin B is also involved in many other physiological processes such as inflammation, wound repair, apoptosis, and activation of thyroxine and renin [2]. Cathepsin L is present in all mammalian tissues and degrades both exogenous and endogenous proteins [3]. Cathepsin L also regulates MHC class II antigen presentation by cleaving Ii, especially in the thymic endothelium where it generates epitopes required for T-cell selection [3,4]. Due to the many immune system processes that these enzymes regulate, alteration in their function could be detrimental to the host.

Nanotechnology is a growing field of study focused on the development of materials sized <100 nm. These nano-sized particles exhibit unique physical and chemical properties that are often different than the bulk material from which they are derived [5]. Nanomaterials are being adapted for many uses, but until recently not much effort has been put toward examining the biological effects of these unique materials. Recent studies have demonstrated that silver nanoparticles (Ag-NPs), which are receiving considerable attention due to their antimicrobial properties, are actually quiet toxic to mammalian cells at low doses [6-8]. Bulk and nanosilver have been shown to interact readily with mammalian proteins and inactivate cellular enzymes, which could contribute to Ag toxicity in vitro [9-11]. In this current study, we examined the interaction of Ag-NPs with cathepsins B and L and determined the extent of enzymatic inhibition caused by the nano-Ag using both purified cathepsins and a cell culture model. The effects of gold nanoparticles (Au-NPs) on cathepsin activity were also assessed due to recent concerns involving the effects of cathepsin L on functionalized Au-NPs [12]. Au-NPs are generally found to be non-toxic to mammalian cells in culture [13], and there is little evidence indicating how they interact with enzymes and whether they alter enzymatic function.

## Materials and Methods

### Nanoparticles

Plasma gas-synthesized 25-nm Ag-NPs were a kind gift from Dr. Karl Martin (Novacentrix, Austin, TX). Colloidal Ag-NPs at 10 nm and Au-NPs at 10 or 30 nm were synthesized by Dr. Steven Oldenburg (NanoComposix, San Diego, CA). Polysaccharide-coated Ag-NPs (PS-Ag) 10 and 25 nm were a generous gift from Dr. Dan Goia (Clarkson University, Center for Advanced Materials Processing, Potsdam, NY). These PS-Ag NPs were synthesized by the reduction of Ag ions in solution by a polysaccharide (acacia gum), which leads to a polysaccharide surface coating [8]. All NPs were well characterized in our laboratory and were diluted in deionized water to 1 mg/ml, sonicated with a probe sonicator, and then further diluted in PBS or cell culture media.

### Cell Line

Vero cells were obtained from the ATCC (CCL-81; Manassas, VA). The Vero cells were maintained in Dulbecco's modified Eagle's Medium (DMEM; Biowhitaker, Basel, Switzerland) supplemented with 10% heat-inactivated fetal bovine serum (FBS; Gibco, Carlsbad, CA) and 1% penicillin-streptomycin (PS; Invitrogen, Carlsbad, CA).

### Biocompatibility Assay

Vero cells were seeded into 96-well tissue culture-treated plates at a concentration of 50,000 cells/well. Twenty-four hours post-seeding, the Vero cells were exposed to Ag- or Au-NPs. The NPs were diluted to the various doses in DMEM + PS and sonicated for 30 s prior to exposure. At 24-h post-exposure, a standard MTS assay (Promega, Madison, Wisconsin) measuring mitochondrial function was performed to determine cell viability.

### Cellular Cathepsin Activity Assay

Vero cells were treated with the above NPs at various doses and were incubated for 24 h at 37°C in 5% CO_2_. The NPs were removed and excess NPs were washed off with PBS. CV-(RR)_2_ or CV-(FR)_2_ reagents were added to the treated cells (diluted 1:250 in PBS) to detect changes in cathepsin B or cathepsin L activity, respectively (Biomol International, Plymouth Meeting, PA). Biomol's cathepsin detection kit utilizes the fluorophore, cresyl violet (CV), linked to two peptides moieties (arginine-arginine for cathepsin B and phenylalanine-arginine for cathepsin L), which are cleaved when the enzyme is active to release the fluorophore. The cathepsin substrate was incubated with the cells for 45 min at 37°C in 5% CO_2_ and was then read using a fluorometer (BioTeck Synergy HT with Gen5 software) at excitation/emission wavelengths of 594/625 nm. Alternatively for the imaging, Hoechst (Molecular Probes – Invitrogen, Carlsbad, CA) was added directly to the CV-(RR)_2_ or CV-(FR)_2_ reagents (1:1,000), and the cells were imaged following the 45-min incubation with the Becton Dickinson Pathway 435 spinning disc Confocal microscope (BD, Franklin Lakes, NJ). The CV-(RR)_2_ or CV-(FR)_2_ reagents were detected with a rhodamine filter with settings normalized to the control (untreated) sample.

### Purified Cathepsin Activity Assay

Purified cathepsins B and L were purchased from Biomol International (SE198 and SE201, respectively). Three micrograms of each protein was incubated with the 4 types of Ag-NPs at either 10 or 50 μg/mL in 300 μL sterile H_2_O for 1 h at room temperature with rotation. Following this incubation, the mixture was added in triplicate to a 96-well black-sided plate (100 μL/well). Hundred microliters of the respective Biomol CV reagent (diluted 1:125 in water) was then added to each well of the enzyme-NP mixture. The reaction was incubated for 45 min at 37°C in 5% CO_2_ and was then read in a fluorometer. The process was repeated to obtain a total of six replicates.

### Statistical Analysis

Data were expressed as the mean ± standard error of the mean (SEM). The one-way ANOVA (Prism 5 statistical and graphing software; GraphPad Software, Inc, La Jolla, CA) was used for the data analysis. The one-way ANOVA was used to determine the effect of test compound concentration on the mean number of treatments/well. *P* ≤ 0.05 was used as the level for significance.

## Results

### Ag-NP Biocompatibility in Vero cells

**Figure 1:**
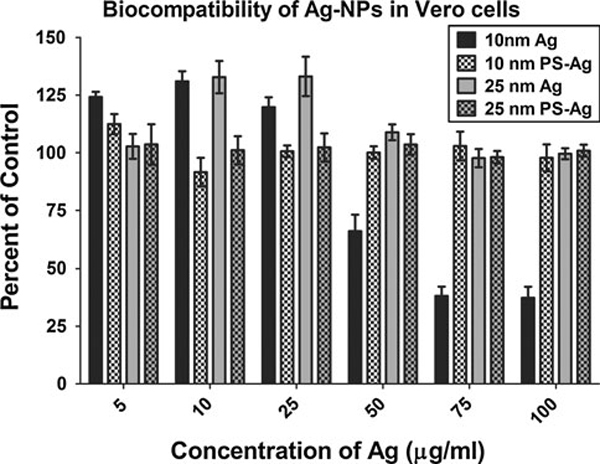
**Biocompatibility of Ag-NPs in Vero cells**. Cytotoxic levels were determined for uncoated and polysaccharide-coated 10 and 25-nm Ag-NPs, following a 24-h exposure using a standard MTS cell viability assay. The cell viability in the treatment groups is expressed as percent control and plotted as the mean +/- standard error of the mean (SEM). (*n* = 8).

After a 24-h exposure, a 25% decline in cell viability was observed in Vero cells exposed to 50 μg/ml of 10-nm uncoated Ag-NPs (Figure 1). Treatments with 10-nm uncoated Ag-NPs at 75 and 100 μg/ml resulted in a 60% reduction in cell viability (Figure 1). There was no further reduction in cell viability in the 50 μg/ml dose, but the cells treated with 75–100 μg/ml died off by day 2 (data not shown). Concentrations of uncoated 10-nm Ag-NPs lower than 50 μg/ml had little effect on Vero cell viability (Figure 1). The 10-nm PS-Ag had no significant effects on the Vero cells in the first 24 h (Figure 1), but the 75 and 100 μg/ml doses demonstrated a 25% reduction in viability after 48 h (data not shown), suggesting an instability of the coating. The concentrations of Ag-PS 10 nm at 50 μg/ml or less had no effect on cell viability at later time points (data not shown). There was little cytotoxicity observed in Vero cells treated with the uncoated or polysaccharide-coated 25-nm Ag-NPs (Figure 1).

### Cathepsin B Activity in Ag-NP-treated Cells

**Figure 2:**
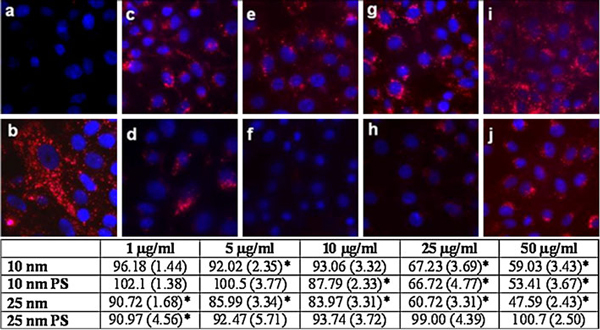
**Cathepsin B confocal imaging in Ag-NP-treated Vero cells**. A fluorescent substrate cleaved by active cathepsin B was detected using confocal microscopy in Vero cells treated with Ag-NPs or left untreated. **a** Negative control (Vero cells alone), **b** Positive control (Vero cells + CV-(FR)_2_), **c** 10-nm uncoated Ag-NP 10 μg/ml, **d** 10-nm uncoated Ag-NP 50 μg/ml, **e** 10-nm PS-Ag-NP 10 μg/ml, **f** 10-nm PS-Ag-NP 50 μg/ml, **g** 25-nm uncoated Ag-NP 10 μg/ml, **h** 25-nm uncoated Ag-NP 50 μg/ml, **i** 25-nm PS-Ag-NP 10 μg/ml, **j** 25-nm PS-Ag-NP 50 μg/ml. Red fluorescent intensity was normalized to Vero cells exposed to the substrate (**b**). The table below represents a quantitative assessment of the confocal images as determined using a fluorescent plate reader. The values indicate the mean percent of control +/- SEM (*n* = 6).

A significant decrease in red fluorescent intensity, indicating a decrease in cathepsin B activity, was observed in the 50 μg/ml doses of 10 nm both uncoated and PS-coated and 25-nm uncoated Ag-NPs (Figure 2d, f, h) over the untreated control (Figure 2b). There was little visual difference in red fluorescence intensity between the 10 μg/ml treated groups (Figure 2c, e, g, i) and the 25-nm PS-Ag at 50 μg/ml (Figure 2j) from the untreated control (Figure 2b), although the 10-nm PS-Ag and 25-nm uncoated Ag-NPs did have a significant decline in fluorescence intensity (Figure 2 table). The decrease in cathepsin B activity in Vero cells treated with Ag-NPs was confirmed via fluorescent quantification in a fluorescent plate reader and a dose-dependent decrease in cathepsin B activity is observed in all treatment groups except for the 25-nm PS-Ag, which interestingly had no effect on cathepsin B activity (Figure 2 table).

### Cathepsin L Activity in Ag-NP-treated Cells

**Figure 3:**
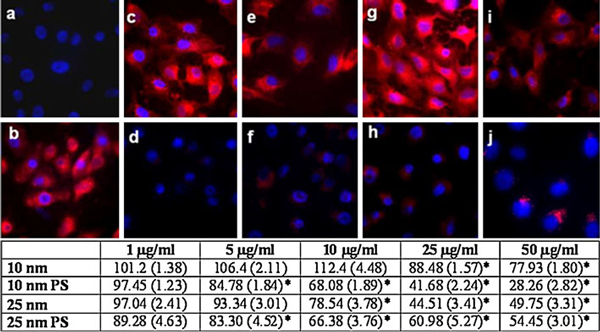
**Cathepsin L confocal imaging in Ag-NP-treated Vero cells**. A fluorescent substrate cleaved by active cathepsin L was detected using confocal microscopy in Vero cells treated with Ag-NPs or left untreated. **a** Negative control (Vero cells alone), **b** Positive control (Vero cells + CV-(FR)_2_), **c** 10-nm uncoated Ag-NP 10 μg/ml, **d** 10-nm uncoated Ag-NP 50 μg/ml, (**e**) 10-nm PS-Ag-NP 10 μg/ml, **f** 10-nm PS-Ag-NP 50 μg/ml, **g** 25-nm uncoated Ag-NP 10 μg/ml, **h** 25-nm uncoated Ag-NP 50 μg/ml, **i** 25-nm PS-Ag-NP 10 μg/ml, **j** 25-nm PS-Ag-NP 50 μg/ml. Red fluorescent intensity was normalized to Vero cells exposed to the substrate (**b**). The table below represents a quantitative assessment of the confocal images as determined using a fluorescent plate reader. The values indicate the mean percent of control +/- SEM (*n* = 6).

Cathepsin L activity appears to be more sensitive to Ag-NP exposure. All 4 types of Ag-NPs tested demonstrated a significant reduction of cathepsin L activity in Vero cells (Figure 3). Minimal cathepsin L activity was observed when the cells were treated with any of the Ag-NPs at 50 μg/ml (Figure 3d, f, h, j), and this decrease was determined by quantitative assessment to be statistically significant (Figure 3 table). There was little discernable difference in red fluorescence between the 10 μg/ml uncoated Ag-NP-treated cells (Figure 3c, g) versus the untreated control (Figure 3b), however, the PS-Ag-NPs (10 and 25 nm) appear to cause a slight, yet significant decline in cathepsin L activity at this dose (Figure 3e, i, table). The decrease in cathepsin L enzymatic activity caused by Ag-NPs was much greater than that observed with cathepsin B (Figures 2, 3).

### Effects of Ag-NPs on Purified Cathepsins

**Figure 4:**
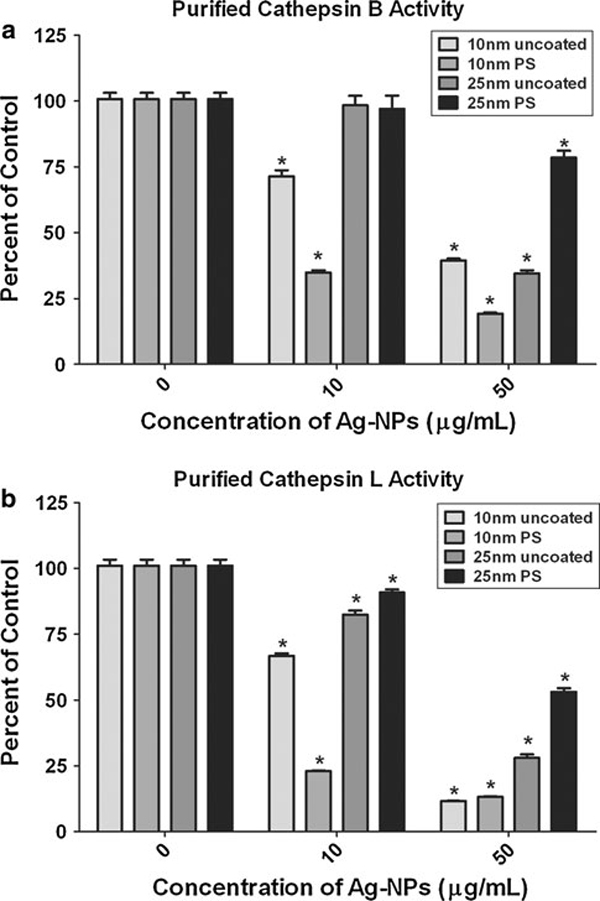
**Ag-NP effects on purified cathepsin B and L enzymes**. Ag-NPs were incubated with purified cathepsin B (**a**) or L (**b**) enzymes. The amount of cathepsin activity in the treatment groups is expressed as percent control and plotted as the mean +/- SEM. Fluorescence was determined using a fluorescent plate reader (*P < 0.05, *n* = 6).

To determine whether the Ag-NPs were influencing the cell signaling or the enzyme directly, the Ag-NPs were also incubated with purified cathepsin B or L, which were then assayed for activity. A significant decrease was seen in the activity of the purified cathepsin B enzyme with the 10-nm Ag-NPs at 10 μg/ml and even greater at 50 μg/ml, regardless of the presence or absence of the PS coating (Figure 4a). The 25-nm uncoated Ag-NPs had little effect on cathepsin B activity at 10 μg/ml, but exhibited similar affects as the 10-nm particles at a dose of 50 μg/ml (Figure 4a). The 25-nm PS-Ag displayed a similar effect as the 25-nm uncoated Ag-NP, with no effect at 10 μg/ml and a significant decrease in cathepsin B activity at 50 μg/ml, however, the decrease with this particle was much less than that with the uncoated 25-nm Ag-NP (Figure 4a). When the purified cathepsin L enzyme was treated with Ag-NPs, the effect on activity was also much more significant than the effect on purified cathepsin B (Figure 4). All four Ag-NPs tested caused a significant decrease in cathepsin L activity at both the 10 and 50 μg/ml doses, with the 10-nm Ag-NPs (uncoated and PS-coated) causing a more pronounced effect (Figure 4b).

### Au-NP Biocompatibility in Vero Cells

**Figure 5:**
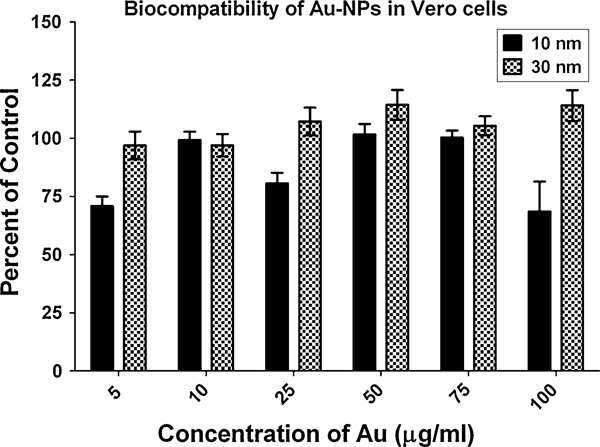
**Biocompatibility of Au-NPs in Vero cells**. Cytotoxic levels were determined for 10- and 30-nm Au-NPs, following a 24-h exposure using a standard MTS cell viability assay. The cell viability in the treatment groups is expressed as percent control and plotted as the mean +/- SEM (*n* = 8).

The Au-NPs at 10 and 30 nm had minimal effects on the viability of Vero cells in culture (Figure 5). There was a slight decrease in the mitochondrial function of the Vero cells treated with very low (5 μg/ml) or very high (100 μg/ml) doses of Au-NP 10 (Figure 5), but a recovery was made by the cells following a 48-h exposure (data not shown).

### Au-NP effects on Cathepsin Activity

**Figure 6:**
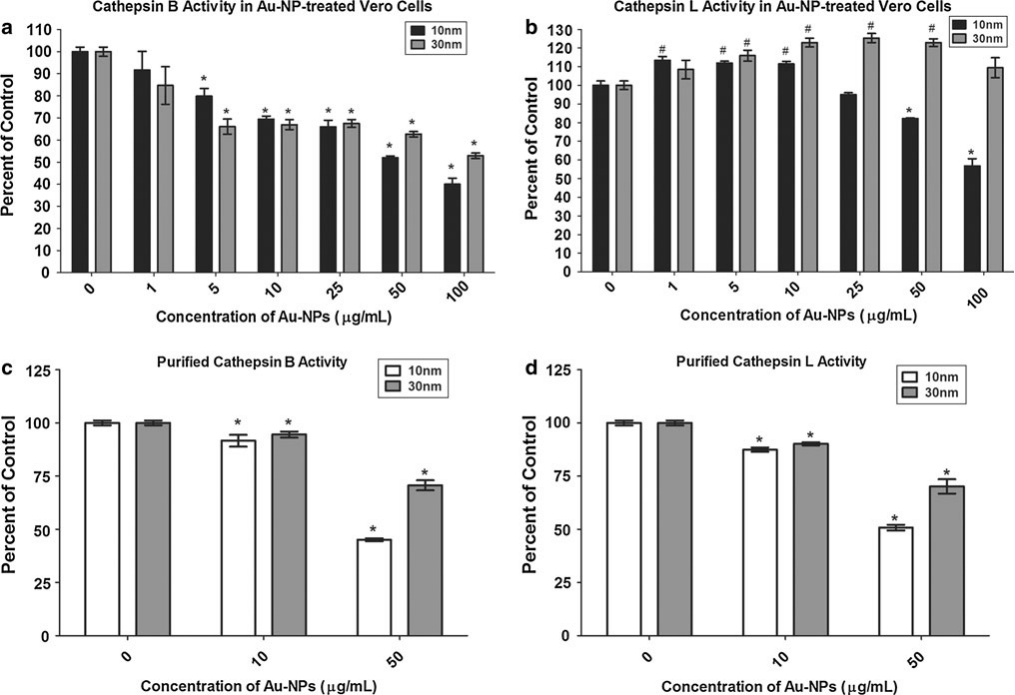
**Quantitative assessment of cathepsin activity following Au-NP exposure**. A fluorescent substrate was processed by cathepsin B (**a**) or cathepsin L (**b**) in Vero cells or purified cathepsin B (**c**) or L (**d**) treated with Au-NPs or left untreated. The fluorescence released following proteolytic cleavage of the substrate was determined using a fluorescent plate reader. The amount of cathepsin activity in the treatment groups is expressed as percent control and plotted as the mean +/- SEM. (*P < 0.05 significantly reduced, #*P* < 0.05 significantly increased; *n* = 6).

After a 24-h exposure of Vero cells to Au-NPs, a slight decrease in cathepsin B activity was observed in cells treated with either 10- or 30-nm Au-NPs at doses of 5–25 μg/ml (Figure 6a). A more significant decrease in cathepsin B activity was observed when the Vero cells were exposed to higher doses of these NPs (Figure 6a). Conversely, the low doses of 10- and 30-nm Au-NPs actually had a stimulatory effect on cathepsin L activity (Figure 6b). At concentrations of 1–10 μg/ml of the 10-nm particles and 5–50 μg/ml of the 30-nm particles, a significant upregulation of cathepsin L was observed in Vero cells (Figure 6b). A significant decrease in cathepsin L activity was finally observed at 50–100 μg/ml of the 10-nm Au-NP, but no decrease was ever seen in Vero cells treated with 30-nm Au-NPs (Figure 6b). Interestingly, incubation of the Au-NPs with the purified cathepsin enzymes had little impact on the enzymatic activity of either cathepsin B or L at 10 μg/ml (Figure 6c, d), and although there was a significant decrease in the 50 μg/ml dose of Au-NPs (Figure 6c, d), the decline in cathepsin activity with these NPs was not as dramatic as that observed with the Ag-NPs (Figure 4).

## Discussion

The research illustrated here demonstrates the effects of Ag- and Au-NPs on cathepsin enzymatic function. We have shown that Ag-NPs decrease the efficiency of cathepsin cleavage in a dose-dependent manner with significant effects observed at doses as low as 1–5 μg/ml, which is non-toxic to the cell itself. The inhibitory effects of the NPs on cathepsin activity were observed within as little as 1 h of incubation (data not shown); following 24 h of incubation with the NPs, the cathepsin activity was significantly affected in Vero cells. The Ag-NPs had a greater effect against the activity of purified cathepsins than that of Vero cell cathepsins. This may suggest a possible direct interaction of the Ag-NPs with the proteins; however, the internalized Ag-NPs are likely to interact with many proteins in the cell and not just the cathepsins, therefore diluting the dose actually exposed to the cathepsins themselves, which could also account for this difference. Bulk silver and Ag-NPs have previously been shown to inhibit the sodium-potassium ATPase [9,10] and FYN kinase [11], respectively, indicating that Ag inhibits different types of enzymatic function, which should be considered prior to use in living systems. Au-NPs also inhibited cathepsin B activity both in a cellular model and with the purified enzyme. However, even more interesting was the impact of Au-NPs on cathepsin L activity. When the Vero cells were exposed to Au-NPs, a stimulatory effect of cathepsin L activity was observed. Conversely, a decline in cathepsin L activity was observed when the Au-NPs were incubated with the purified enzyme. This may indicate that the Au-NPs are interacting with the cathepsins indirectly, possibly through a signaling cascade, inside of the cells.

The biological applications of both Au- and Ag-NPs are very promising, but little effort has been put into determining how they may affect the natural function of the cells in which they are incorporated. Both Ag- and Au-NPs are capable of recognizing and readily binding thiol groups of proteins and other ligands, suggesting that they would likely have similar effects on protein activity [14,15]. However, the two types of nanomaterials have substantially different effects on cytotoxicity. Au-NPs are considered to be relatively benign to most cells, which make them an attractive tool for biomedical applications [13]. Conversely, Ag-NPs have been shown to be toxic to cell lines at low concentrations [8] and appear to have a more significant affect on altering enzymatic activity.

Biological molecules are being added to Au- and Ag-NPs to improve their biocompatibility or functionality for biological applications. However, recently, it was determined that cathepsin L can cleave biological moieties off functionalized nanoparticles [12]. See et al utilized functionalized gold nanoparticles to assess the ability of cathepsin L to cleave substrates off the NPs and confirmed that upon internalization into the cell, the substrate is cleaved off the Au-NPs by cathepsin L [12]. We utilized similar sized Au-NPs and Ag-NPs to determine the effects of these NPs on cathepsin activity. We concluded that the Au-NPs, but not the Ag-NPs, stimulated cathepsin L activity in cells, confirming that functional groups added to the surface of Au-NPs may be lost upon cell internalization. Alternatively, cathepsin B activity was decreased by both types of NPs testing, indicating that cathepsin B directed cleavage of biomolecules will be inhibited in the presence of NPs. Our findings suggest that the use of cathepsin L inhibitors or alternate core metals may be necessary for use of intracellular nano-based therapeutics and sensors.

Cathepsin activity is important for many cellular processes, most notably for priming of the adaptive immune response through antigen processing [16]. Therefore, using materials that are capable of inhibiting cathepsin activity for biological applications may perhaps cause harmful side-effects. Studies have suggested the potential for Ag-NPs as an antimicrobial: however, if they cause a dramatic reduction in cellular cathepsin activity, which can alter the adaptive immune response, they may actually interfere with the host's ability to clear the infection. There are a lot of beneficial biomedical applications for using nanomaterials, but careful consideration to avoid potential undesired effects must be determined before they are used in vivo.
